# Effects of Lactate on One Class of Group III (CT3) Muscle Afferents

**DOI:** 10.3389/fncel.2020.00215

**Published:** 2020-08-06

**Authors:** Rochelle A. Peterson, Christine König, Katharina Zimmermann, Christine M. Barry, Lukasz Wiklendt, Simon J. H. Brookes

**Affiliations:** ^1^Neurogastroentrology Laboratory, Flinders Health and Medical Research Institute, College of Medicine and Public Health, Flinders University, Adelaide, SA, Australia; ^2^Klinik für Anästhesiologie am Universitätsklinikum Erlangen, Friedrich-Alexander Universität Erlangen-Nürnberg, Erlangen, Germany; ^3^Musculoskeletal Neurobiology Laboratory, Flinders Health and Medical Research Institute, College of Medicine and Public Health, Flinders University, Adelaide, SA, Australia

**Keywords:** lactate, lactic acid, ergoreceptor, HCAR1, metaboreceptor

## Abstract

A class of Group III muscle afferent neurons has branching sensory terminals in the connective tissue between layers of mouse abdominal muscles (“CT3 muscle afferents”). These sensory endings are both mechanosensitive and metabosensitive. In the present study, responses of CT3 afferents to lactate ions and changes in temperature were recorded. Raising muscle temperature from 32.7°C to 37°C had no consistent effects on CT3 afferent basal firing rate or responses to either von Frey hair stimulation or to an applied load. Superfusion with lactate ions (15 mM, pH 7.4) was associated with an increase in firing from 6 ± 0.7 Hz to 11.7 ± 6.7 Hz (14 units, *n* = 13, *P* < 0.05, *P* = 0.0484) but with considerable variability in the nature and latency of response. Reducing the concentration of extracellular divalent cations, which mimicked the chelating effects of lactate, did not increase firing. Raised concentrations of divalent cations (to compensate for chelation) did not block excitatory effects of lactate on CT3 afferents, suggesting that effects *via* ASIC3 were not involved. Messenger RNA for the G-protein coupled receptor, hydroxyl carboxylic acid receptor 1 (HCAR1) was detected in dorsal root ganglia and HCAR1-like immunoreactivity was present in spinal afferent nerve cell bodies retrogradely labeled from mouse abdominal muscles. HCAR1-like immunoreactivity was also present in axons in mouse abdominal muscles. This raises the possibility that some effects of lactate on group III muscle afferents may be mediated by HCAR1.

## Introduction

Striated muscle is innervated by multiple classes of sensory neurons with a range of properties. Specialized muscle spindles give rise to rapidly conducting Group I and II fibers. Group III and IV fibers are smaller in diameter, with slower conduction and encode mechanosensitive, metabosensitive and nociceptive signals from muscle. A class of group III mechanosensitive afferents was recently characterized in detail and shown to have endings in the connective tissue layers closely associated with muscle fibers, and were both mechanosensitive and metabosensitive. These were referred to as “CT3” afferents. They had saturating responses to low levels of muscle stretch and could be potently activated by a metabolite mix comprising adenosine triphosphate (ATP), lactate, and low pH (Peterson et al., 2019). The signaling mechanisms of both ATP and low pH have been well characterized. ATP responses are largely mediated *via* P2X receptors; P2X2 and P2X3 receptors in particular are located on many sensory endings (Burnstock and Williams, 2000; Reinöhl et al., 2003; Burnstock et al., 2013; Bernier et al., 2018). In contrast, pH changes are often detected in sensory endings *via* ASIC channels, particularly ASIC3 (Molliver et al., 2005; Naves and McCleskey, 2005; Gregory et al., 2015).

How lactate contributes to the potent responses evoked by the metabolite mix is unknown. Lactate is released in large quantities by active muscle and in lesser amounts by a wide range of cells throughout the body (Hall et al., 2016; Brooks, 2018; Ferguson et al., 2018). In striated muscle, lactate is mostly formed by glycolytic conversion of glucose to pyruvate, which is then converted into lactate, with the production of an H+ ion. Since its discovery in 1780, lactate has often been portrayed as a metabolic waste product, associated with muscle fatigue and oxygen debt (Ferguson et al., 2018). However, lactate production occurs in both the presence and absence of oxygen (Brooks, 2018), giving rise to the cell-cell shuttle theory (Brooks, 1985, 2007) in which it plays multiple roles in whole-body metabolism. In the nervous system, lactate has been variously proposed to be involved in glial-neuron shuttling, glial to neuron signaling (Tang et al., 2014), as a substrate of neural metabolism (Pellerin and Magistretti, 1994; Hertz, 2004; Bergersen, 2007), as a volume transmitter (Lauritzen et al., 2014), in cell signaling (Liu et al., 2009; Lauritzen et al., 2014), in volume regulation (Halestrap, 2013) and modulation of affect (Carrard et al., 2018). In terms of sensory biology, lactate may contribute to pain sensitivity by actions on spinal nociceptors (Hasegawa et al., 2018).

The basis of these effects of lactate in the nervous system, and particularly on primary afferent neurons, are currently unclear. Lactate injected into the arterial blood supply had little effect on afferent firing (Rotto and Kaufman, 1988). However, infusion of lactate with protons and/or ATP into muscle evoked noxious sensations in human subjects and withdrawal responses in rats (Pollak et al., 2014; Gregory et al., 2015). In combination, these 3 metabolites (ATP, lactate, and protons) modified intracellular Ca^2+^ signals and response properties in isolated spinal afferent nerve cell bodies (Light and Perl, 2003; Light et al., 2008; Jankowski et al., 2013).

While lactate ions had limited effects on some muscle afferents (Rotto and Kaufman, 1988) lactic acid may excite other afferents (Sinoway et al., 1993). Effects may be mediated by lactate’s ability to chelate calcium and magnesium ions in solution. Decreases in divalent cation availability enhance responses to protons by ASIC3 channels (Immke and McCleskey, 2001; Molliver et al., 2005; Naves and McCleskey, 2005). Thus, it has been suggested that the effects of lactate on afferent firing may be mediated indirectly by the chelation of divalent cations. Whatever the mechanism, there is good evidence that metabolites act synergistically, when applied in combination (Light et al., 2008; Pollak et al., 2014; Gregory et al., 2015). It is also possible that a fourth product of active muscles, temperature, may influence muscle afferents (Hertel et al., 1976; Racinais et al., 2017).

Understanding the detailed physiological properties of thin fiber muscle afferents is important, as mechano- and metabo-sensitive muscle afferents stimulate the exercise pressor reflex (Alam and Smirk, 1937). The exercise pressor reflex increases mean arterial pressure, cardiac output and ventilation during exercise in animals and humans (Alam and Smirk, 1937; Coote et al., 1971; McCloskey and Mitchell, 1972). Group III and group IV muscle afferents make up the sensory arm of the pressor reflex (McCloskey and Mitchell, 1972; Kaufman et al., 1984). Group III muscle afferents were suggested to be largely responsible for responses to mechanical stimuli while group IV afferents responded to metabolites (Hayes and Kaufman, 2001; Kaufman and Hayes, 2002). However, some group III afferents also respond to metabolites released by working muscles and therefore, potentially play a role in evoking the metabolic component of the exercise pressor reflex (Rotto and Kaufman, 1988).

In this study, we tested whether CT3 (group III) afferents were responsive to lactate ions alone and whether this was likely to be mediated by changes in the availability of divalent cations. We also explored whether the hydroxyl carboxylic acid receptor 1 (HCAR1, also called GPR81) for which lactate is an endogenous ligand (Cai et al., 2008; Liu et al., 2009) could be involved. HCAR1 is expressed in many tissues including white and brown fat (Offermanns et al., 2011) heart, stomach, intestine, and skeletal muscle, and has been reported in neurons in the hippocampus, and cerebellum (Liu et al., 2009). Lastly, we tested whether CT3 afferents were activated by a raised temperature, in the range occurring in active muscle.

## Materials and Methods

### Ethical Approval

Male and female C57Bl/6 mice (*n* = 37), from 4 to 8 weeks old, were bred in-house (Flinders University). Animals had *ad libitum* access to food and water and were euthanized by a lethal dose of isoflurane by inhalation, followed by removal of the heart or decapitation, following Australian Code for the Care and Use of Animals for Scientific Purposes (8th Edn., 2013, National Health and Medical Research Council of Australia) and were approved by the—Flinders University Animal Welfare Committee permits #890/15, #911/16.

### Isolation of Skeletal Muscle-Nerve Preparation

The abdominal wall musculature (external oblique, internal oblique, transversus abdominis, and rectus abdominis) was excised. The external oblique and rectus abdominis muscles were then removed, leaving a preparation of internal oblique and transversus muscle which was pinned in a petri dish lined with Sylgard (Dow Corning, Midland, MI, USA), containing Krebs solution (in mM: NaCl, 118; KCl, 4.75; NaH_2_PO_4_, 1.0; NaHCO_3_, 25; MgCl_2_, 1.2; CaCl_2_, 2.5; glucose, 11; bubbled with 95% O_2_/5% CO_2_) and warmed to 37°C.

### Anterograde Labeling

The preparation was transferred to a purpose-built chamber for anterograde labeling. The dissected end of the nerve trunk was led into a side-chamber filled with paraffin oil and sealed with silicone grease and a coverslip. A drop of 5% biotinamide solution (Molecular Probes, Eugene, OR, USA) in “artificial intracellular medium” (150 mM monopotassium L-glutamic acid, 7 mM MgCl_2_, 5 mM glucose, 1 mM EGTA, 20 mM HEPES, 5 mM disodium adenosine-triphosphate, 0.02% saponin, 1% dimethyl sulphoxide) was placed onto the de-sheathed nerve trunk under the paraffin. The main chamber was then filled with sterile culture medium (Tassicker et al., 1999) and incubated for 4 h on a rocking tray in a humidified incubator at 37°C, 5% CO_2_ in air. The preparation was then fixed overnight in modified Zamboni’s fixative in a separate dish at 4°C. Preparations were permeabilized (see below) and biotinamide was visualized with streptavidin-Cy3 (1:500, Molecular Probes, 4 h). Preparations were then immunohistochemically labeled (see below), washed in PBS, and mounted in 100% carbonate-buffered glycerol (pH 8.6). They were mounted between two coverslips attached to a 1 mm thick aluminum surround which could be viewed from either side.

### Immunohistochemistry

#### Tissue Fixation and Processing

Samples used for immunohistochemistry were fixed in modified Zamboni’s fixative (0.2% saturated picric acid in 2% paraformaldehyde in 0.1 mol L-1 phosphate buffer, pH 7.2) for 24 h. The tissue was then washed repeatedly in phosphate-buffered saline (PBS; 3 × 10 min), permeabilized and cleared with dimethyl sulphoxide (DMSO; 2 × 10 min, 1 × 30 min) and rinsed in PBS (3 × 10 min). The samples were then treated with Triton X-100 (0.5%) in PBS on a mixing tray at room temperature overnight. They were then rinsed in PBS, and further permeabilized in 100% glycerol with 0.1% sodium azide, on an orbital mixer (OM5, Ratek, VIC, Australia) in a humidified incubator at 37°C for 24 h then washed with PBS solution (3 × 30 min) to remove the glycerol. Preparations of abdominal muscle (*n* = 5) were then incubated with primary antisera at room temperature for 2 days. The HCAR1 antibody, GPR81 s296 (Cat# SAB1300090; Sigma–Adrich, St. Louis, MO, USA), was raised in rabbit against the N-terminal extracellular domain of HCAR1(1:200)). Preparations were also co-labeled with anti-calcitonin gene-related peptide (CGRP; Abcam, Cambridge, MA, USA, raised in sheep, 1:500) and anti-neurofilament 200 (NF200, Sigma–Aldrich, St. Louis, MO, USA, raised in mouse, 1:1,000). Preparations were rinsed three times in PBS and incubated with the appropriate secondary antisera for 4 h at room temperature, all raised in donkey and obtained from Jackson ImmunoResearch (West Grove, PA, USA). Details of antibody combinations are summarized in Table 1. After final rinses with PBS, preparations were mounted in carbonate-buffered glycerol (pH 8.6).

**Table 1:** List of primary and secondary antibodies.

Primary Antibody	Source	Species	Dilution	Secondary Antisera	Source	Dilution
GPR81/HCAR1	Sigma	Rabbit	1:200	Donkey Anti-Rabbit CY3	Jackson	1:400
CGRP	Abcam	Sheep	1:500	Donkey Anti-Sheep CY5	Jackson	1:200
NF200	Sigma	Mouse	1:1,000	Donkey Anti-Mouse AMCA	Jackson	1:100

#### Sections of Dorsal Root Ganglion

Dorsal root ganglia were immersion-fixed in modified Zamboni’s fixative (*n* = 5, T11 to L1) then placed in PBS containing 3% sucrose as a cryoprotectant overnight before being embedded in O.C.T (Optimal Cutting Temperature) compound (Tissue-Tek, Torrance, CA, USA). The ganglia were cut into 12 μm thick sections on a cryostat (Leica-Riechert Cryocut 1800 Cryostat, Germany) at −19°C and thaw-mounted onto polyethyleneimine-coated slides. Every third section was collected on the same slide to avoid double counting and approximately 24 sections were counted for each ganglion. Slides were allowed to dry overnight at room temperature and subsequently stored at 4°C, protected from light. Normal donkey serum (10%) for 30 min was used as a blocking agent. Antisera against HCAR1, CGRP, and NF200 were then applied to sections. The sections were incubated in primary antisera at room temperature overnight. The preparations were then washed with PBS (3 × 10 min) and incubated with appropriate secondary antibodies (Table 1) for 2 h. After washing with PBS preparations were mounted as described.

### Close Extracellular Single-Unit Recordings

Several nerve trunks (2–4 trunks) entering the preparation were mobilized for 5–8 mm. Preparations were transferred to a 4 ml, Sylgard-lined recording chamber, and the dissected nerve trunks were pinned, under slight tension, to the Sylgard, using 50 μm tungsten pins. The chamber was continuously superfused with warmed, oxygenated Krebs solution (at 30°C). A paraffin oil bubble was attached to the base of the chamber, and the end of the dissected nerve trunk was then pinned inside the oil bubble. Recordings were made from the nerve with a 100 μm Pt/Ir wire electrode insulated apart from the last 400 μm. Signals were amplified (ISO-80, WPI, Sarasota, FL, USA), recorded at 20–40 kHz (PowerLab16s/p, LabChart 7 Pro software, AD Instruments, Sydney, NSW, Australia). Single units were discriminated by amplitude and duration using Spike Histogram software (AD Instruments, Sydney, NSW, Australia). Further offline discrimination of units was carried out using an in-house program that combined amplitude/duration discrimination with principal component analysis. Spikes were identified by a voltage threshold and for each identified peak, 10 voltage values were obtained at 100 μs intervals (over 1 ms in total), creating an individual spike signal. These spike signals were stacked into a matrix with one row per spike and principal component analysis was performed on this matrix. Scores from the principal components were then used as features in a Gaussian mixture model to separate distinct clusters; each corresponding to a separate neuron. These could be compared in the same software to clusters derived from amplitude/duration discrimination. Results are presented from 32 afferent recordings (*n* = 22), in which 19 afferents were positively identified as CT3 afferents (Peterson et al., 2019), nine demonstrated muscle spindle characteristics, and four units could not be identified.

#### Identifying CT3 Afferents

CT3 afferents were positively identified by using criteria, previously described in Peterson et al. (2019), these included the typical characteristics of low threshold saturating responses to von Frey hairs and to stretch by applied load, under isotonic conditions.

##### Focal Compression

With the muscle under minimal resting load (~0.5 g), mechano-transduction sites were identified by probing the surface of the preparation with a 1.0 mN von Frey hair, evoking bursts of action potentials. Responsive sites were marked on the tissue, using fine carbon particles applied *via* the tip of the von Frey hair. Once a receptive field was marked in this way, a range of von Frey hairs (0.2, 0.49, 1.0, 1.96, and 4.9 mN) was applied to the receptive field to test whether responses tended to saturate (which was another defining feature of CT3 afferents).

##### Static Stretch Responses

A purpose-built 8 mm array of hooks was used to connect one edge of the preparation, *via* a cotton thread, to an isotonic transducer (52–9511, Harvard Bioscience, South Natick, MA, USA) with a (minimal) resting load of 0.5 g. Stretches were applied by adding counterweights (1–6 g) for 30 s to the isotonic lever; these constant loads stretched the muscle evenly across its width, while the transducer recorded changes in length. A minimum rest period of 3 min was allowed between stretches.

#### Thermal Stimulation of Muscle Afferents

For 17 afferents (*n* = 9), the temperature of the superfusing Krebs solution was changed in a “ramp-like” manner from ~32.7°C to ~37.0°C by switching between two heating pumps which pre-warmed the Krebs solution before it entered the bath, similar to a previously described counter-current temperature exchange system (Winter et al., 2017). The basal afferent activity was recorded at 32.7°C for 60 s, then identified afferents were probed with a 100 mg von Frey hair. After a further 60 s, a 2 g load was applied to the preparation for 30 s and a further period of 120 s was allowed for recovery. The Krebs solution was then raised to 37.0°C and the same sequence of mechanical stimuli were applied again. The temperature was then returned to 32.7°C and another bout of stimuli delivered. This was repeated until three bouts of low temperature, separated by two periods of raised temperature had been recorded. Bath temperatures were recorded by a custom-made thermocouple (GIR/GIA, GHM GROUP—Greisinger, Regenstauf, Germany), and a commercial temperature probe (Cat# MLT415/D, AD Instruments, Sydney, NSW, Australia) at two points in the recording chamber.

#### Application of Lactate

Sodium lactate (15 mM) was applied by superfusion to 13 preparations. After identifying CT3 afferents in standard Krebs solution, the superfusate was switched to HEPES-buffered solution (139 mM NaCl; 4.7 mM KCl; 1.2 mM MgCl_2_; 11 mM Glucose; 5 mM HEPES; and 2.5 mM CaCl_2_, pH 7.4) and allowed to equilibrate for 15 min at 4 ml/min. The bathing solution was then switched to HEPES buffered solution containing 15 mM sodium lactate; with NaCl reduced to compensate osmotically (i.e., 124 mM NaCl; 4.7 mM KCl; 1.2 mM MgCl_2_; 11 mM Glucose; 5 mM HEPES; and 2.5 mM CaCl_2_) at pH 7.4). The pH of the lactate solution was adjusted to 7.4 using 300 mM HCl. The lactate-containing solution was perfused for 15 min, then replaced with the HEPES solution for a further 15 min. Thus, exposure to lactate was achieved without changes in pH, osmolarity, or temperature. The HCAR1 agonist 3,5-dihydroxybenzoic acid (300 μM; Liu et al., 2012) was similarly applied by superfusion for 10 min.

#### Application of Low Calcium and Magnesium Ion Solution

To test whether chelation of calcium and magnesium ions mediated effects of lactate, we used a mimic solution (Immke and McCleskey, 2001; Naves and McCleskey, 2005) based on the free ion concentrations in lactate solutions from published equilibrium constants (Martell and Smith, 1977; Immke and McCleskey, 2001). The “mimic” solution had reduced concentrations of both calcium and magnesium ions (130 mM NaCl, 5 mM KCl, 0.88 mM MgCl_2_, 10 mM Glucose, 5 mM HEPES, 1.77 mM CaCl_2_, pH 7.0) matching the reduction in free ions caused by chelation by 15 mM lactate. In these studies, preparations (*n* = 6) were equilibrated in a HEPES solution containing (130 mM NaCl, 5 mM KCl, 1 mM MgCl_2_, 10 mM Glucose, 5 mM HEPES, 2 mM CaCl_2_) for 15 min then exposed to the “mimic” solution for 15 min. They were then washed out for 30 min with the original solution. Preparations were then exposed for 15 min to a modified 15 mM lactate solution containing increased concentrations of calcium and magnesium ions (115 mM NaCl, 5 mM KCl, 1.12 mM MgCl_2_, 5 mM HEPES, 2.35 mM CaCl_2_, 15 mM lactate) to exactly compensate for chelation (Immke and McCleskey, 2001). In this way, we tested whether the chelation of divalent cations was likely to contribute to the activation of CT3 afferents by 15 mM lactate.

### RNA Isolation and Quantitative Real-Time PCR

Quantitative real-time PCR was used to detect mRNA for HCAR1 in the freshly dissected hippocampus, gastrocnemius muscle, dorsal root ganglia (T9-L1), and abdominal adipose tissue of C57BL/6 mice (*n* = 4). Tissues were harvested and placed into Trizol (Sigma), and then homogenized using a Tissue lyser (Qiagen, Melbourne, VIC, Australia). RNA was extracted using the Direct-zol RNA mini prep- with on-column DNase treatment (Zymo-Spin ICC Columns, Zymogen, Irvine, CA, USA) according to the manufacturer’s instructions. RNA purity and concentration were determined using a Nanodrop 2000 (Thermo Fischer Scientific, Scoresby, VIC, Australia). cDNA was produced from the isolated RNA using IScript Reverse Transcription Supermix according to the manufacturer’s protocols (Superscript II, Biorad, Australia). Real-time PCR was performed using the StepOnePlus cycler (Life Technologies, Scoresby, VIC, Australia). Target-genes were identified and amplified using predesigned Taqman primers and probes (Cat#4331182, Thermo Fischer Scientific, Scoresby, VIC, Australia) to HCAR1, the samples were normalized to β-actin and GAPDH (see Table 2). A sample with no cDNA template served as the negative control, with all samples run in triplicate. Primer efficiencies were determined for each target. Resultant PCR products were separated on a 2% SB agarose gel with a 100 bp DNA ladder (Invitrogen Scoresby, VIC, Australia, Cat# 15628-019) and imaged using the Gel Doc EZ Imager (Bio-rad, Gladesville, NSW, Australia) and Image Lab software (Bio-rad). Target gene expression was determined relative to the endogenous controls using Q-Gene analysis software (Simon, 2003).

**Table 2:** TaqMan primer assays.

Primer	Accession no.	Amplicon length (bp)	Reference
GPR81/ HCAR1	Mm00558586_s1	82	Lauritzen et al. (2014)
β-actin	Mm02619580_g1	143	Liu et al. (2009)
GAPDH	Mm99999915_g1	109	Lauritzen et al. (2014)

### Retrograde Labeling

Retrograde labeling combined with immunohistochemistry was used to test whether HCAR1-like immunoreactivity was expressed by spinal sensory neurons that innervate the abdominal muscles (external and internal obliques, transversus abdominis). Male and female C57BL/6 mice (*n* = 4) were anesthetized by isoflurane inhalation (induced at 4%, maintained at 1.5% in O_2_) and a 10 mm long incision was made on the ventral abdomen, approximately 15 mm lateral from the midline, to expose the abdominal wall musculature on the right-hand side. Five microliters of cholera toxin subunit B (CTxB), conjugated with Alexa Fluor 488 (1 mg/ml; Molecular Probes, VIC, Australia), was applied over three sites *via* a glass micropipette (outer diameter: 1.5 mm, inner diameter: 1.12 mm; Cat# TW 150-4; World Precision Instruments, WPI) with a tip diameter of approximately 5 μm using a custom-made N_2_ pressure-ejection system (Biomedical Engineering, Flinders University, 1 s duration @ 0.3 Hz; 70 kPa). Following tracer injection, the skin was closed using 5.0 sutures (Dynek, Australia) and OpSite spray (Cat# 66004978; Smith and Nephew, Hull, UK). Mice recovered for at least 7 days and were then euthanized by isoflurane overdose, followed by removal of the heart. The abdominal muscles (internal and external obliques and transversus abdominis), dorsal root ganglia (T9-L1), spinal cord, and skin surrounding the incision were removed then fixed and permeabilized as previously described. DRG’s (T9-T11) were sectioned, as described above. To avoid double-counting, every fourth section of DRGs was collected on the same slide. Out of these four slides, every second slide was processed with antisera for HCAR1, CGRP, and NF200.

### Microscopy and Image Processing

Immunohistochemically stained preparations were viewed and analyzed on an Olympus IX71 inverted fluorescence microscope, fitted with appropriate dichroic mirrors and filters. Images of labeled neural structures were captured with a CoolSNAP ES digital camera (Roper Scientific, Photometerics, Tucson, AZ, USA) using analySIS 5.0 software (Soft Imaging System, Gulfview Heights, South Australia, SA, Australia). Image processing was restricted to brightness and contrast adjustments, cropping, and formation of photomontages using Adobe Photoshop (CS6, Adobe Systems Inc., San Jose, CA, USA).

### Statistical Analysis

Statistical comparisons were performed using Prism 7 software (GraphPad Software, Inc., San Diego, CA, USA). Student’s paired *t*-tests were to compare the effects of applied solutions in electrophysiological studies. To investigate possible differences in cell size in immunohistochemistry studies, one-way ANOVAs were used, with corrections for multiple comparisons. To investigate the abundance of HCAR1 mRNA in qPCR studies, one-way ANOVAs were used, with corrections for multiple comparisons. Differences were considered significant if *P* < 0.05. Results are expressed as means ± 95% confidence intervals except when stated otherwise. The number of animals used in each set of experiments is indicated with “n.” NS denotes a non-significant finding (*P* > 0.05).

## Results

### Identifying CT3 Muscle Afferents

**Figure 1:**
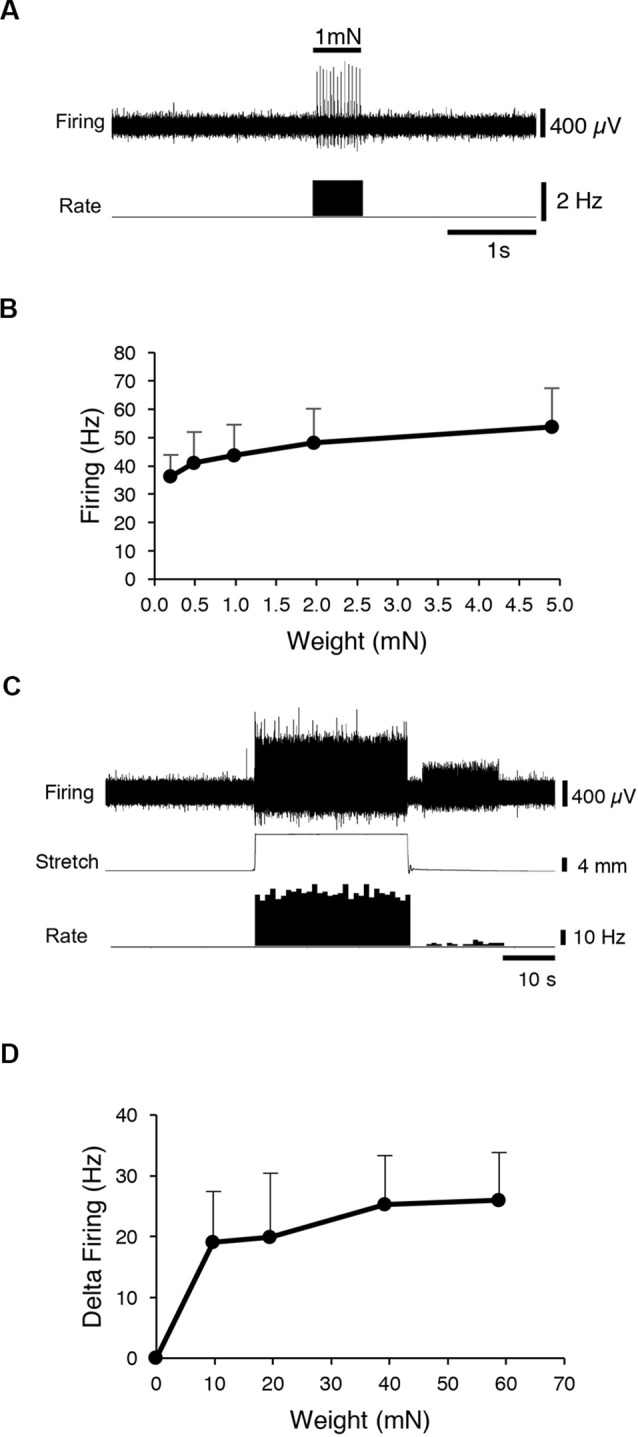
Identification of CT3 afferents. CT3 afferents, recorded from segmental nerves to abdominal muscles were identified by their responses to graded von Frey hairs and responses to stretch. **(A)** Typical responses to 100 mg von Frey hair. **(B)** Mean results of a von Frey hair stimulus-response curve for 14 units. **(C)** Typical tonic response to a 2 g load. **(D)** Mean responses to a range of loads applied to the preparation (14 units, *n* = 13. These responses are similar to those reported previously for CT3 afferents (Peterson et al., 2019).

First, CT3 muscle afferents were identified by their characteristic, saturating responses to graded von Frey hair probing and to stretch (Peterson et al., 2019). A 1 mN von Frey hair was applied (units 14, *n* = 13), to map the mechanosensitive receptive field of each unit. Then a range of von Frey hairs (0.2, 0.49, 0.98, 1.96 and 4.9 mN) was applied with minimal resting tension, evoking maximum instantaneous firing of 53.8 ± 13.7 Hz (14 units, *n* = 13; Figures 1A,B) very similar to our previous study (Peterson et al., 2019). All preparations (units 14, *n* = 13) were then stretched by imposed loads of 1–6 g. Each load evoked a dynamic response at the onset, with firing maintained at an elevated rate throughout the stretch, with modest adaptation (Figures 1C,D). These properties are all consistent with units belonging to CT3 muscle afferents described recently (Peterson et al., 2019). Several other units, with different responses, were also recorded, some of which were identified as muscle spindle afferents by the location and orientation of their elongated receptive fields.

### CT3 Muscle Afferent Response to 15 mM Lactate in HEPES

**Figure 2:**
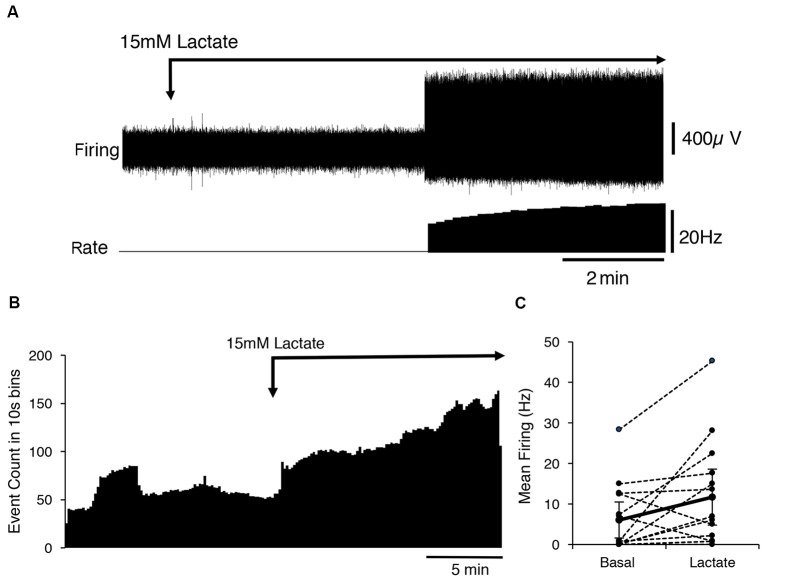
Activation of CT3 afferents by superfusion with 15 mM Lactate (pH 7.4). **(A)** A typical example of the effects of the application of a 15 mM lactate solution; The Firing rate of a single identified CT3 unit is shown on the lower trace. The unit did not fire spontaneously but after several minutes of perfusion of lactate, there was an abrupt start of firing at about 15 Hz, which increased in frequency over the next few minutes. **(B)** The mean response of 14 discriminated CT3 units is shown during the application of 15 mM lactate solution, averaged over 15 min in 10 s bins (14 units, *n* = 13). Note the large sustained increase in spontaneous firing in the presence of lactate. **(C)** Comparison of basal firing (black dots) averaged over 15 min before and after the application of lactate for 14 identified CT3 units. Most units show an increase in firing in lactate, although two units showed a marked decrease in firing rate.

Fourteen identified CT3 muscle afferents were exposed to 15 mM lactate by superfusion (*n* = 13). On average, 15 mM lactate superfusion evoked a small but significant increase in firing across all 14 units. Average basal firing was 6 ± 0.7 Hz and in the presence of 15 mM lactate this increased to an average of 11.7 ± 6.7 Hz (14 units, *n* = 13, *P* < 0.05, *P* = 0.0484, *t* = 2.178, *df* = 13, paired *t*-test; Figure 2). Overall, average firing peaked (16 ± 9 Hz, *n* = 14) more than 10 min after the first arrival of lactate in the bath. However, responses were highly variable in both timing and amplitude, and spontaneous bursts of firing complicated analysis. Of the 14 units exposed to 15 mM lactate, three units responded within 10 s of lactate arriving in the bath, while the others started to respond up to 5 min later. Of the 14 units, eight units showed a clear increase in firing in lactate; four units showed no detectable change (Figure 2), and two units had large bursts of spontaneous firing during the control period and showed an overall decrease in firing in 15 mM lactate.

### The Response of CT3 Muscle Afferents to HCAR1 (GPR81) Agonist 3, 5-Dihyroxybenzoic Acid (3,5-DBHA)

3,5-Dihydroxybenzoic acid (300 μM, 3,5-DHBA) is an HCAR1 agonist (Liu et al., 2012). The application of 3,5-DHBA evoked no change in the firing rate of CT3 afferents. The average firing rate in 3,5-DHBA was 16 ± 13 Hz compared to the average basal firing 14 ± 10 Hz, indicated no differences (Seven units, *n* = 5, *P* > 0.05, *P* = 0.05057, *t* = 0.7077, *df* = 6 paired *t*-test). Application of 3,5-DHBA *via* superfusion caused preparations to contract markedly; this was presumed to be an off-target effect as the contraction was not seen with even the highest concentration of lactate.

### The Response of CT3 Muscle Afferents to Low Calcium and Magnesium Ion “Mimic” Solution

**Figure 3:**
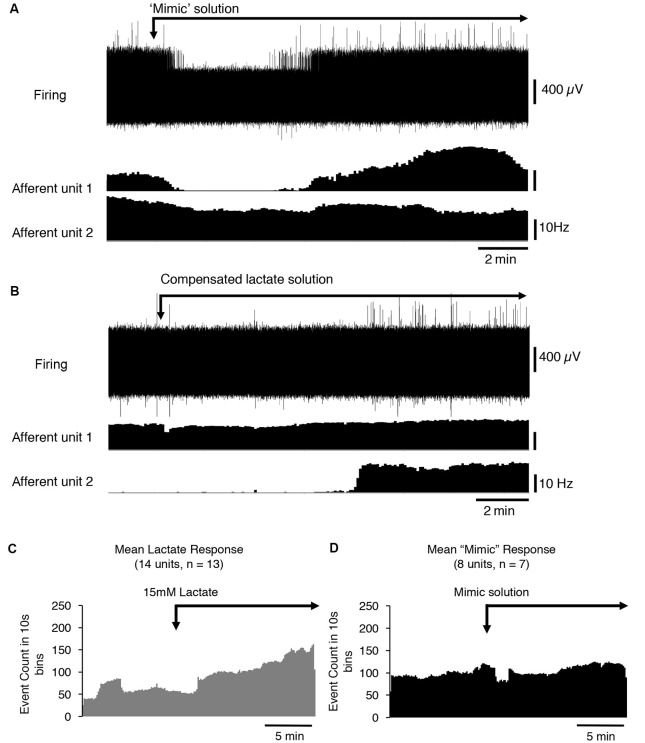
The response of CT3 afferents to low Ca^2+^ and Mg^2+^ “mimic” solution and compensated lactate solution. **(A)** Responses of two identified CT3 afferents recorded in a single nerve to the application of a “mimic” solution’ containing reduced concentrations of Ca^2+^ and Mg^2+^ ions, which mimicked the chelating effects of lactate on divalent cations. One CT3 afferent (afferent unit 1) initially decreased, then increased firing after switching to the mimic solution. Afferent unit 2 showed a small, gradual decrease in the firing. Overall there was no significant effect of the mimic solution (*P* = 0.7431, *t* = 0.3411, *df* = 7, paired *t*-test). **(B)** The application of 15 mM lactate, with raised [Mg^2+^] and [Ca^2+^] (to compensate for chelation effects) modestly activated unit 1 and was associated with a larger effect, after a delay, in Unit 2. This suggests that the effects of lactate are not due to the reduction in the availability of the divalent cations. **(C)** The mean response of 14 identified CT3 units, (*n* = 13) to 15 mM lactate applied by superfusion in normal buffered solution (same as Figure 2B–included here for comparisons). **(D)** Mean response of eight units (*n* = 7) to the “mimic” solution with reduced [Ca^2+^] and [Mg^2+^] to emulate the chelating effects of 15 mM lactate. Note that the “mimic” solution did not replicate the effect of 15 mM lactate shown in **(C)**.

Lactate chelates calcium and magnesium ions in solution and thus reduces their available concentrations. This increases the sensitivity of the acid-sensing ion channel, ASIC3 to H+ ions and has been suggested to mediate the effects of lactate (Immke and McCleskey, 2001). We tested whether reducing [Ca^2+^] and [Mg^2+^] to mimic their free concentration in 15 mM lactate (Immke and McCleskey, 2001) affected CT3 afferent firing similarly to lactate. Of eight CT3 afferents tested (*n* = 7), five showed no response to the reduced calcium and low magnesium HEPES solution, one showed an increase in firing, and two units had a decrease in firing (Figure 3A). Overall, there was no significant response of CT3 muscle afferents to the low calcium and low magnesium HEPES solution (average basal firing 9.7 ± 8.7 Hz, average response 10.9 ± 10.3 Hz, *P* < 0.05, *P* = 0.7431, *t* = 0.3411, *df* = 7, paired *t*-test Figures 3C,D). This suggests that lactate does not solely act on CT3 muscle afferents *via* the chelation of Ca^2+^ and Mg^2+^ ions.

After the mimic solution had been washed out, we then applied a 15 mM lactate solution in which [Ca^2+^] and [Mg^2+^] were raised above control levels ([Ca^2+^] = 2.35 mM and [Mg^2+^] = 1.12 mM), so that free [Ca^2+^] and [Mg^2+^] matched those in lactate-free HEPES solution. If the reduced activity of the divalent cations was responsible for lactate effects; this compensated solution should be without effect. Figure 3B shows that lactate still excited CT3 muscle afferents under these conditions, supporting the idea that lactate was not working solely through the chelation of calcium and magnesium ions. Of the eight units recorded, five units showed an increase in firing in the presence of 15 mM lactate with raised [Ca^2+^] and [Mg^2+^]. Three units did not respond to the 15 mM lactate under these conditions.

### Thermal Stimulation of Muscle Afferents

To determine whether muscle afferents encoded temperature, a further 17 units (*n* = 9) were recorded. Most of the units (13/17) had no spontaneous firing during the basal period at 32.7°C and this did not change as the temperature was raised to 37°C. Four units had spontaneous firing at 32.7°C. One was characterized as a CT3 afferent; the other three units belonged to muscle spindle afferents, which had an average spontaneous firing rate of 22 Hz ± 14 Hz. As bath temperature was increased from 32.7°C, all four units initially increased spontaneous firing rate. This was then followed by a decrease below baseline firing and ceasing firing altogether above 35°C. The firing did not recover when the temperature was subsequently lowered. None of the recorded afferents demonstrated a sustained increase in firing rate when the temperature was raised to 37°C.

#### Interactions Between Thermal and Mechanical Stimuli

**Figure 4:**
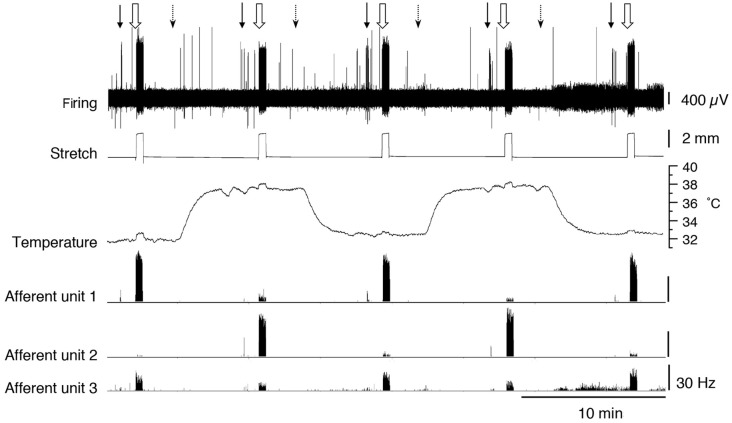
Firing of three muscle afferent units [spontaneous, von Frey hair-evoked (arrows) and stretch-evoked (open arrows)] and effects of temperature (dotted arrows). Three different responses were seen among three units discriminated from the same nerve. Afferent unit 1 had no spontaneous firing and responses to both von Frey hairs and stretch were decreased at 37°C. Afferent unit 2 also lacked spontaneous firing but showed larger responses to both von Frey hairs and stretch at 37°C compared to 32.7°C. Afferent unit 3 had modest spontaneous firing which was reduced at the raised temperature. Unit 3’s responses to von Frey hairs and stretch were both reduced at 37°C.

We tested whether warming caused changes in excitability that were not reflected in the basal firing rate. When tested with a von Frey hair (1 mN) during thermal stimulation, 5 of 17 units (*n* = 9), showed an increase in peak instantaneous firing rate at raised temperature (37°C) and 10 units had a decreased peak response. The other two units were inconsistent. When tested with stretch, using a 2 g load, 16 of 17 units showed a reduction in stretch-evoked firing at the higher temperature. One unit demonstrated an increase in the stretch-evoked firing rate (Figure 4). Of the 17 units, four were classified CT3 afferents, nine were identified as muscle spindle afferents and four could not be identified. Overall, average responses to von Frey hairs and to stretch by load were all decreased at 37°C. However, averaged firing to von Frey hairs of the 4 identified CT3 afferents at 37.0°C were 37.1 ± 26.5 Hz compared to 13 ± 8.75 Hz at the basal temperature of 32.7°C; this was not significant (four units, *n* = 4, *P* > 0.05, *P* = 0.1848, *t* = 1.716, *df* = 3 paired *t*-test, see Table 3).

**Table 3:** Interactions thermal and mechanical stimuli.

	Basal firing (Hz)		Von frey hair [IFR (Hz)]		Stretch [Delta firing rate (Hz)]	
Classification	32.7°C	37.0°C	32.7°C	37.0°C	32.7°C	37.0°C
CT3 (*N* = 4)	38.0 (one of four units)	0.0 ± 0.0	13.0 ± 8.75	37.1 ± 26.5	13 ± 8.4	1.8 ± 1.0
Muscle Spindle (*N* = 9)	22.0 ± 13.9	2.1 ± 3.9	43.8 ± 14.4	21.1 ± 15.4	34.3 ± 15.8	9.6 ± 11.6
Unclassified (*N* = 4)	0.0 ± 0.0	0.0 ± 0.0	13.3 ± 10.6	26.1 ± 37.1	18.6 ± 29.2	9.8 ± 16.7

*Summarizes properties of afferents at two temperatures: 32.7°C and 37°C, in terms of basal firing, peak instantaneous firing evoked by a 1 mN von Frey hair and change in firing (compared to basal) evoked by 2 g load, according to fibre type*.

### HCAR1 Immunohistochemistry

#### Presence of HCAR1-Like Immunoreactivity in Dorsal Root Ganglia

**Figure 5:**
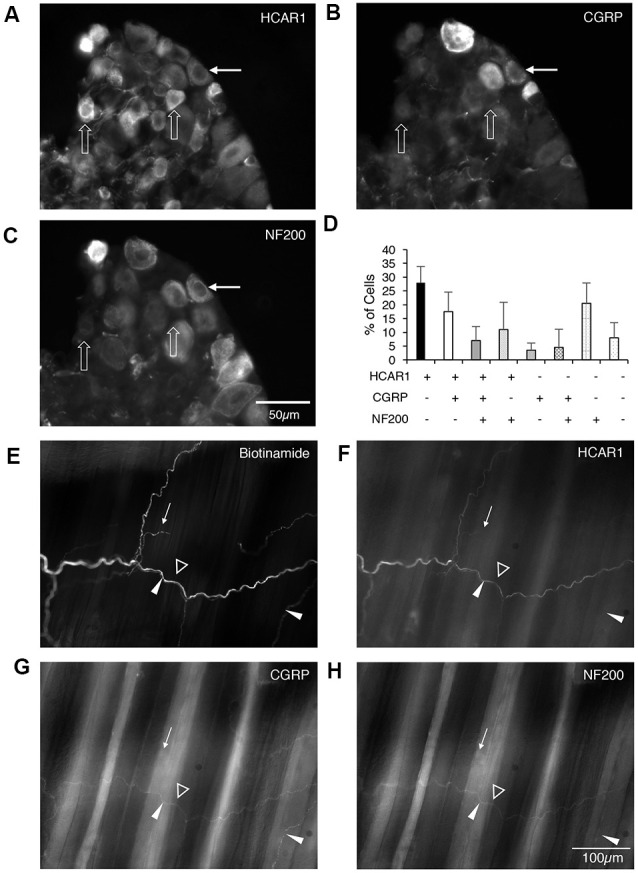
Localization of hydroxyl carboxylic acid receptor 1 (HCAR1), CGRP, and NF200 in dorsal root ganglia (T10-L1) and abdominal muscles. **(A–C)** Thoracolumbar dorsal root ganglion immunohistochemically labeled for HCAR1, CGRP, and NF200. HCAR1-like immunoreactivity was present in small and medium cell bodies which largely lacked CGRP and NF200 (open arrows). A slightly larger cell had low-intensity immunoreactivity for HCAR1 and was immunoreactive for CGRP and NF200 (white arrows). **(D)** The proportion of nerve cell bodies with various combinations of the 3 markers is shown (200 cells; *n* = 4). **(E)** A bundle of axons and some single axons branching in the connective tissue between abdominal muscle layers were revealed by biotinamide tracing with autofluorescent striated muscle bundles visible in the background. HCAR1 was present in some of the axons in bundles (open arrowhead), and also in a single axon (white arrow). **(F)** HCAR1-immunoreactive axons ran alongside CGRP immunoreactive axons in bundles (**G**; white arrowheads) but were not located in the same axons. **(H)** Some axons in biotinamide-labeled bundles were also labeled with NF200 antiserum (open arrowhead).

HCAR1-like immunoreactivity was detected in dorsal root ganglion neuronal cell bodies; the labeling was cytoplasmic, non-nuclear, and was not associated with the cell membrane. It occurred with a wide range of intensities and, in many cases, the initial axon process was clearly labeled. Satellite cells in dorsal root ganglia were not labeled. T11–T13 DRG nerve cell bodies were quantified for HCAR1, CGRP, and NF200 immunoreactive content in triple-labeled 12 μm sections (*n* = 4). HCAR1-like immunoreactivity was detected in 63.5 ± 8% of all T11–T13 DRG cell bodies (*n* = 4). Of all neurons sampled, 28 ± 6% were immunoreactive for HCAR1 alone (i.e. HCAR1+/CGRP−/NF200−); 17.5 ± 7% were HCAR1 co-localised with CGRP but lacking NF200 (HCAR1+/CGRP+/NF200−), 11 ± 10% were HCAR1 co-localised with NF200 (HCAR1+/CGRP−/NF200+), and 7 ± 5% were immunoreactive for all three neurochemical markers (HCAR1+/CGRP+/NF200+; Table 4 and Figures 5A–D). HCAR1-like immunoreactivity coexisted with other markers in populations of neurons with a variety of soma sizes: HCAR1+/CGRP−/NF200− immunoreactive neurons had an average soma size of 186 ± 24 μm^2^, HCAR1+/CGRP+/NF200− cell bodies were similar at 184 ± 32 μm^2^ (*n* = 4, NS *P* > 0.05, *P* = 0.9182, *df* = 19, one-way ANOVA), HCAR1+/CGRP+/NF200+ were medium-sized cells at 420.36 ± 49 μm^2^ (*n* = 4, *P* < 0.05 *P = 0.0132, *df* = 19, one-way ANOVA) and HCAR1+/CGRP−/NF200+ labeled cells were similarly medium in size with an average of 366 ± 126 μm^2^ (*n* = 4, NS, *P* > 0.05, *P* = 0.539, *df* = 19, one-way ANOVA; Table 4 and Figure 5).

**Table 4:** Presence of HCAR1-like immunoreactivity in dorsal root ganglia.

Soma size	186 ± 24 μm^2^	184 ± 32 μm^2^	366 ± 126 μm^2^	420 ± 49 μm^2^	297 ± 9 μm^2^	364 ± 37 μm^2^	513 ± 30 μm^2^	313 ± 82 μm^2^
% of cells	28 ± 8%	17.5 ± 7%	11 ± 10%	7 ± 5%	3.5 ± 3%	4.5 ± 7%	20.5 ± 7%	8 ± 6%
HCAR1	+	+	+	+	−	−	−	−
CGRP	−	+	−	+	+	+	−	−
NF200	−	−	+	+	−	+	+	−

*Cell size ± standard deviation (and percentage of all neurons sampled) is shown for the eight possible combinations of HCAR1, CGRP, and NF200 antisera. Cells that lacked all three markers were still detectable by autofluorescence. Counts are based on *n* = 4*.

#### Presence of HCAR1 in Whole Mounts of Abdominal Muscles

Nerve trunks innervating the abdominal muscles were anterogradely labeled with biotinamide followed by HCAR1, CGRP, and NF200 antisera. HCAR1-immunoreactive nerve fibers mostly had smooth axons (Figures 5E–H) and did not appear either beaded or varicose. Within large nerve trunks, HCAR1 labeled medium diameter axons which often also co-labeled with NF200. These axons were also smooth rather than varicose (Figure 5F,H).

HCAR1 labeling was also present in nerve fibers in small nerve bundles throughout the muscles itself, as well as in the connective tissue layers. Within the connective tissue and muscle, HCAR1-like immunoreactivity was present in narrow, smooth axons (Figure 5F). These often ran alongside fine varicose axons, some of which were immunoreactive for CGRP. HCAR1 labeling also faintly labeled fat cells. Other structures within these preparations, such as motor endplates and muscle spindles, were not immunoreactive for HCAR1.

#### Retrograde Tracing From the Abdominal Muscles

**Figure 6:**
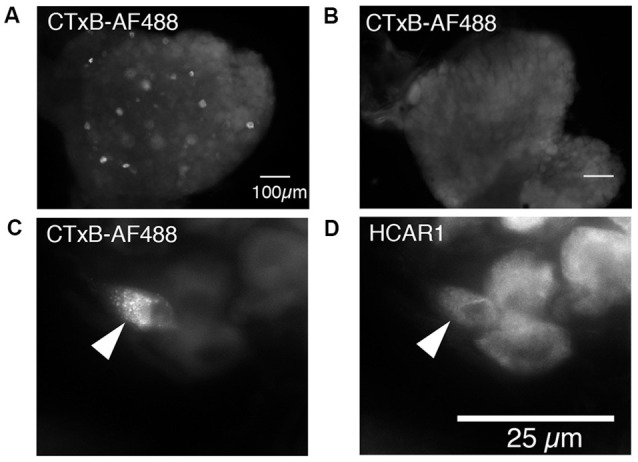
Nerve cell bodies in T11 mouse dorsal root ganglia retrogradely labeled from abdominal muscles; immunohistochemical labeling for HCAR1. **(A)** Nerve cell bodies retrogradely labeled with CTxB-488 applied to the ipsilateral abdominal muscle group are shown in the dorsal root ganglion of T11. Panel **(B)** shows the contralateral ganglion which did not contain labeled neurons. **(C)** Intense CTxB-AF488 labeling in a single nerve cell body was immunoreactive for HCAR1 (**D**—arrows).

Cholera Toxin Subunit B, conjugated to Alexa Fluor 488 (CTxB-AF488), was unilaterally injected into the abdominal muscles to retrogradely label DRG nerve cell bodies (*n* = 4). After 7 days of recovery, retrogradely labeled nerve cell bodies were detectable in ipsilateral but not contralateral thoracic and lumbar DRG (T9 to L1; Figures 6A,B). The number of CTxB-AF488 labeled cell bodies in DRG was counted in wholemount preparations of ganglia. On average, after the application of CTxB-AF488 to three sites in the abdominal muscles, 63 ± 23 neurons were labeled per animal (*n* = 4). These were located in a slightly skewed distribution extending from T9 to L1, with the majority of the cells located between T10 and T12. T11 DRG (*n* = 4) contained the largest number of CTxB-488 labeled cells and were sectioned and labeled with antisera against HCAR1, CGRP, and NF200. The proportion of CTxB-488 labeled cells containing immunoreactivity for the different combinations of markers was quantified. CTxB-488-containing cell bodies that co-localized HCAR1+/CGRP-/NF200- accounted for 6/20 cells tested (Figures 6C,D). CT3 afferent neurons have previously been shown to lack CGRP immunoreactivity and would thus be expected to be HCAR1+/CGRP-. HCAR1+/CGRP+/NF200- cells accounted for 3 of 20 sensory neurons labeled by CTxB-488. Half of the 20 retrogradely labeled neurons analyzed (10/20) lacked HCAR1, CGRP, and NF200 immunoreactivities.

### RNA Isolation and Quantitative Real-Time PCR of HCAR1

We compared the expression of mRNA for the GPCR receptor HCAR1, in the dorsal root ganglia, gastrocnemius muscle, hippocampus, and abdominal adipose tissue of mice. A single PCR product was obtained for HCAR1 in each sample. GAPDH and β-actin were used as dual housekeeping genes as both had previously been compared to HCAR1 expression in these tissues (Liu et al., 2009; Lauritzen et al., 2014).

**Figure 7:**
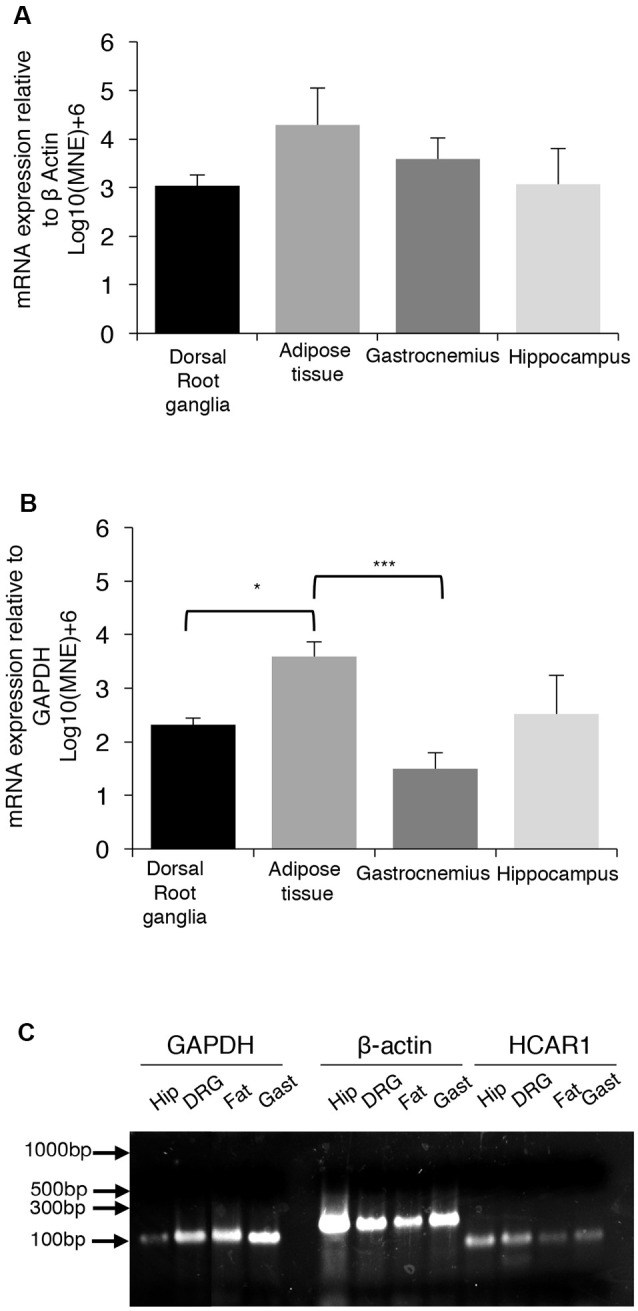
mRNA expression of HCAR1 across tissue samples (*n* = 4). **(A)** Mean expression of HCAR1 normalized relative to β-actin, in dorsal root ganglia (DRG), adipose tissue, gastrocnemius muscle, and brain tissue (hippocampus). Differences in relative expression were not significant. **(B)** The mean expression of HCAR1 normalized relative to GAPDH suggested that content in adipose tissue may be higher than in the DRG and hippocampus. **(C)** Gel separation and molecular weight of PCR products of HCAR1 (~82 bp) for Hip (Hippocampus), DRG (Dorsal root ganglia), Gast (Gastrocnemius) in lanes 9–12. Products for GAPDH (~109 bp) and b-actin (~149 bp) in lanes 1–4 and 5–8, respectively; these are both housekeeping genes commonly used to normalize expression levels. *P < 0.05, ***P < 0.05.

HCAR1 mRNA expression, normalized relative to β-actin, was found to be most abundant in abdominal adipose tissue (4.29 ± 0.75 MNE), followed by the gastrocnemius muscle (3.59 ± 0.43 MNE), hippocampus (3.07 ± 0.73 MNE) and dorsal root ganglia [3.03 ± 0.23 MNE (Figure 7A). However, differences in the abundance of HCAR1 relative to β-actin were not significant between these four tissues (one-way ANOVA corrected for multiple comparisons (*n* = 4, *P* > 0.05, NS, *df* = 11 one-way ANOVA)].

Relative to GAPDH expression, HCAR1 was most abundant in the abdominal adipose tissue (3.59 ± 0.26 MNE), followed by the hippocampus [2.52 ± 0.70 MNE], then dorsal root ganglia (2.32 ± 0.12 MNE)] and gastrocnemius muscle (1.50 ± 0.29 MNE; Figure 7B). There was significantly more HCAR1 mRNA in adipose tissue compared to dorsal root ganglion (*n* = 4, *P* < 0.05*, *df* = 11, one-way ANOVA; Figure 7B) and the gastrocnemius muscle sample (*n* = 4, *P* < 0.05***, *df* = 11, one-way ANOVA; Figure 7B). However, it should be noted that GAPDH is enzymatic and may itself have variable expression between types of tissues. Resultant PCR products separated on a 2% SB agarose gel showed identified bands for each sample, which correspond to the predicted base-pair mass for HCAR1 (82 bp), as well as housekeeping genes GAPDH (109 bp) and β-actin (143 bp; Figure 7C).

## Discussion

### Summary

Targeted extracellular recordings from identified group III muscle afferents (CT3 afferent units) were used to investigate the effects of lactate ions and temperature. CT3 muscle afferents are activated by a mixture of muscle metabolites containing ATP, H+, and lactate (Peterson et al., 2019). Lactate, at a high but physiological concentration (15 mM), activated some CT3 muscle afferents, without the addition of either ATP or protons. Real-time PCR demonstrated the presence of mRNA for the G-protein coupled lactate receptor (HCAR1 or HCAR1) in dorsal root ganglia and HCAR1-like immunoreactivity was detected in cell bodies of dorsal root ganglia neurons that were retrogradely labeled from skeletal muscle. HCAR1-like immunoreactivity was also visible in some nerve fibers in whole-mount preparations of abdominal muscles, including in CGRP-negative axons. The presence of HCAR1+/CGRP- immunoreactive cell bodies and muscle axons is consistent with the expression of HCAR1 by CT3 afferents because CT3 afferents do not express CGRP (Peterson et al., 2019). However, a recent study (de Castro Abrantes et al., 2019) has questioned the specificity of most commercially-available HCAR1 antisera; therefore, we refer to labeling as HCAR1-like immunoreactivity. These data raise the possibility that lactate activates at least one class of sensory afferent endings in skeletal muscle (CT3 afferents) *via* a distinct receptor, which may correspond to the G-protein-coupled receptor, HCAR1. The CT3 afferents do not appear to be sensitive to temperature changes in the range experienced in exercising muscle.

### Lactate and ASIC3

Lactate concentration in skeletal muscle can range from about 1 mM at rest to 30 mM during extreme exercise or ischemia (Ferguson et al., 2018). In the current studies, we demonstrated that CT3 muscle afferents are activated by 15 mM lactate; a moderately high, but physiologically relevant concentration. The effects of lactate on afferents can be mediated *via* indirect effects on ASIC3 channels, since lactate partially chelates calcium and magnesium ions in solution, reducing the availability of these divalent ions in the extracellular fluid (Immke and McCleskey, 2001). The proton binding sites that gate ASIC3 are affected by the reduced availability of calcium and magnesium ions, and this increases their sensitivity to protons (Babini et al., 2002; Birdsong et al., 2010). In isolated dorsal root ganglion cells in culture, lactate appeared to act solely *via* ASIC3 (Immke and McCleskey, 2001). In contrast, the present study recorded the effects of solutions on intact sensory nerve terminals in ex vivo striated muscle preparations. A mimic solution, with free [Ca^2+^] and [Mg^2+^] reduced to the same extent as with 15 mM lactate did not replicate the response to lactate. Furthermore, a solution containing 15 mM lactate with raised [Ca^2+^] and [Mg^2+^] (to compensate for partial chelation) was still effective in exciting some CT3 afferents. Put together, these provide evidence that effects *via* chelation/ASIC3 are not solely responsible for driving lactate-evoked firing in CT3 muscle afferents. This is consistent with studies that have shown that pharmacological blockade of ASIC3 (Tsuchimochi et al., 2011) and genetic knockout of ASIC3 (Kim et al., 2019) have only minor effects on pressor responses to exogenous lactate and mechanical stimulation of the muscle.

### Metabolite Interactions

Sensory neuron responses to acidity and purines have been extensively described since the 1960s. H+ ions often act *via* ASIC channels (Molliver et al., 2005; Gründer and Chen, 2010; Gautam and Benson, 2013), and purines, effects have been attributed to P2X receptors. P2X3 receptors, in particular, are located on many afferent terminals (Burnstock et al., 2013; Fabbretti, 2019). The present study suggests that lactate may also contribute, *via* its specialized receptor, as lactate, applied without ATP or protons, activated many CT3 afferent endings. Osmolarity, pH, temperature, and mechanical artifacts were all controlled during lactate application by superfusion. It should be noted, however, that the preparations consisted of abdominal muscles that had been isolated and dissected, inevitably causing some trauma to the tissue. Damage can cause local acidification (Woo et al., 2004), and possibly the endogenous H+ may have sensitized responses to lactate, although the use of HEPES buffered solution should have minimized this. Studies of the metabolite mix (Light et al., 2008; Jankowski et al., 2013; Pollak et al., 2014) reported that individual components (lactate, H+ and ATP) each had little effect on afferent firing; but demonstrated considerable synergy when applied in combination (Light et al., 2008; Pollak et al., 2014; Gregory et al., 2015). In the present study, interactions between metabolites were not directly studied due to technical constraints: lactate exposure shows marked tachyphylaxis and only a subset of CT3 afferents was lactate-responsive. HCAR1 is a low-affinity receptor, which may allow it to encode across the wide range of lactate concentrations that occur naturally (Liu et al., 2009). Thus, CT3 muscle afferents may act, in part, as lactate “sensors,” while still responding to other stimuli (including mechanical stimuli such as stretch, compression, and other metabolites, but not temperature changes).

### Other Mechanisms of Action of Lactate

Wild-type murine cortical neurons in culture are inhibited by lactate; a response that was absent in HCAR1 knockout mice. The effect was primarily mediated *via* Giα which inhibits adenylyl cyclase but Giβγ- subunits also contributed (de Castro Abrantes et al., 2019). It has also been reported that lactate ions inhibit TRPV1 channels (de La Roche et al., 2016) but this is unlikely to be relevant to CT3 neurons, which do not respond to capsaicin (Peterson et al., 2019) and presumably lack TRPV1. Another mechanism by which nociceptors can be activated by lactic acid as a weak acid involves intracellular acidification acting on TRPA1 (Wang et al., 2011) but it is unlikely that this operates in non-nociceptive group III (CT3) afferents. There have been other reports of excitatory effects of lactate on neurons of the locus coeruleus (Tang et al., 2014), with a relatively low EC50 (680 μM; Bozzo et al., 2013) but the molecular identity of the receptor is uncertain. The results of the present study raise the possibility that HCAR1 is involved in excitation by lactate of group III ergoreceptor CT3 afferents. In the current study, we demonstrated that CT3 afferents are activated by physiological concentrations of lactate. Retrograde labeling and qPCR studies demonstrated that HCAR1, in which lactate is an endogenous ligand, is expressed in dorsal root ganglion neurons that innervate skeletal muscles. Immunohistochemical studies, showed HCAR1-like immunoreactivity is not only present in the dorsal root ganglia, but also axons in the abdominal muscles themselves. HCAR1 immunoreactivity was present in some muscle axons that lacked CGRP immunoreactivity. This is significant as CT3 afferent endings are not CGRP-immunoreactive (Peterson et al., 2019). This all provides circumstantial evidence that HCAR1 may mediate some effects of lactate on CT3 afferents.

### Muscle Afferents and Temperature

In the current study, CT3 afferents did not directly encode temperature. Their mechanosensitivity to von Frey hairs and stretch were not significantly temperature-sensitive either, at least within the range tested. Previous studies investigating the thermosensitivity of muscle afferents have used a larger range of temperatures 0–52°C, in mice, rats, cats, dogs, and humans (Hertel et al., 1976; Mense and Meyer, 1985; Graven-Nielsen et al., 2002; Graven-Nielsen, 2006; Xu et al., 2010; Wilkinson et al., 2012; Jankowski et al., 2013). We studied a smaller, more physiological range, as the intramuscular increases in temperature, as a result of exercise is typically in the range of 2–3°C (Graven-Nielsen, 2006). In humans, the intramuscular temperature increased from 35 to 38°C during exercise (Allsop et al., 1990; Ferguson et al., 2006). Our results were consistent with previous reports that some muscle afferents decrease firing above 35°C (Ottoson, 1965; Hertel et al., 1976; Fischer and Schäfer, 1999). A previous study reported that fewer group III and IV afferents were activated by thermal stimuli than by mechanical stimuli or metabolites (Jankowski et al., 2013). Thermosensitive afferents are often insensitive to innocuous pressure and stretch (Mense and Meyer, 1985). Both findings are consistent with the lack of thermosensitivity in CT3 afferents described here, as this class is sensitive to light pressure, stretch, and metabolites (Peterson et al., 2019).

### Pressor Reflex, Pain, and Muscle Fatigue

The exercise pressor reflex plays an integral role in triggering and maintaining adaptive cardiovascular and hemodynamic responses to exercise (Alam and Smirk, 1937; Mitchell, 2012). At the onset of exercise, the pressor reflex is activated by thinly myelinated (group III) and unmyelinated (group IV) muscle afferents, that are primarily mechanosensitive (group III) or metabosensitive (group IV; McCord and Kaufman, 2009; Secher and Amann, 2012). However, some mechanosensitive group III afferents also show metabosensitivity (McCord and Kaufman, 2009), including responses to lactate (Thimm and Baum, 1987; Rotto and Kaufman, 1988). CT3 afferents are both mechanosensitive (responding to passive muscle lengthening) and metabosensitive to a mixture of mediators (Peterson et al., 2019) and also to lactate alone (as shown in the present study). Since the mechanoreflex component of the pressor reflex can be activated by passive muscle lengthening (Drew et al., 2017) and by intramuscular injections of lactic acid (Rotto and Kaufman, 1988; Sinoway et al., 1993) CT3-like afferents are likely to play a significant role in the exercise pressor reflex. It is unlikely that raised muscle temperature sensitizes these endings because warming was generally associated with a decrease in firing (see above). Muscle warming was reported to reduce the pressor response to exogenous P2x agonists; an effect that may in part reflect reduced axonal excitability (Li and Cui, 2016).

It is interesting to note that in studies of human subjects, stimulation of both group III and group IV afferents were associated with localized cramp-like pain in the muscle (Simone et al., 1994). Many algesic compounds have been shown to sensitize or activate both group III and group IV afferents including bradykinin, potassium ions, arachidonic acid, ATP, and lactic acid (McCord and Kaufman, 2009). However, muscle nociceptors were not activated by innocuous stretch or light compression (Simone et al., 1994) which potently activate CT3 afferents (see Figure 1), suggesting that CT3s may not be involved in nociception.

The pressor reflex underlies the exaggerated cardiovascular responses to exercise associated with heart failure, hypertension, and peripheral artery disease. In these disease states, it has been suggested that exercise pressor reflexes are over-activated. Group III and Group IV muscle afferents have also been implicated in other conditions, including the development of peripheral and central fatigue and chronic fatigue syndrome (Mense and Schiltenwolf, 2010; Amann et al., 2015; Staud et al., 2015; Stone and Kaufman, 2015; Stone et al., 2015; Mitchell, 2017; Sidhu et al., 2018). The contribution of CT3 afferents to these conditions remains to be determined. Group III and group IV muscle afferents activate pathways in the CNS, which limit the output of spinal cord motor neurons i.e.,: they also play a key role in generating central fatigue (Laurin et al., 2015; Sidhu et al., 2017, 2018). Whether CT3 afferents are involved in this pathway is currently uncertain.

### Limitations of the Study

Group IV muscle afferents were not recorded during this study and it is known that some of these are metabosensitive. Local occlusion of the circulation during muscle contraction revealed activation of both group III and IV afferents by metabolic products (Kaufman et al., 1984; Iwamoto et al., 1985), A small proportion of group IV afferents is sensitive to lactate (Kniffki et al., 1978; Darques et al., 1998). A comparison of the effects of lactate on group III and group IV muscle afferents would be valuable to understand their relative contributions to the exercise pressor reflex under normal physiological conditions, as well as in disease. It should be noted that HCAR1 is not the only channel or receptor known to mediate responses to lactate, as mentioned above. ASIC3 may also contribute (Immke and McCleskey, 2001), TRPA1 may act as a detector of weak acids, including lactate (Wang et al., 2011) and TRPV1 is inhibited by lactate ions (de La Roche et al., 2016). A body of circumstantial evidence has been presented that suggests a role for HCAR1 in CT3 afferents. Further investigations would be required to establish this, including studies in HCAR1 knockout mice and tests of receptor antagonists, when these become available.

## Conclusions

In the present study, excitation of CT3 muscle afferents by physiological levels of lactate, without the addition of ATP or protons, was demonstrated. The same class of afferents was not thermosensitive. Activation by lactate was not mediated by the chelation of calcium or magnesium ions. The current study raises the possibility that lactate can activate CT3 afferents *via* a distinct receptor, which may correspond to the G-protein-coupled receptor, HCAR1. Further studies are required to investigate the coexistence of receptors for other metabolites (purine receptors, ASIC channels) with HCAR1 on CT3 afferents. The role of CT3 receptors in the pressor reflex, relative to other classes of muscle afferents, remains to be determined.

## Data Availability Statement

All datasets presented in this study are included in the article.

## Ethics Statement

The animal study was reviewed and approved by Flinders University Animal Welfare Committee permits #890/15, #911/16.

## Author Contributions

Conception and design of the experiments were carried out by RP, CB, KZ, CK, and SB. Collection and assembly of data was by RP and CK. Analysis and interpretation of data was by RP, CB, LW, KZ, CK, and SB. Drafting the article or revising it critically for important intellectual content was by RP, CB, LW, KZ, CK, and SB. All authors have approved a final version of the manuscript.

## Conflict of Interest

The authors declare that the research was conducted in the absence of any commercial or financial relationships that could be construed as a potential conflict of interest.

## Abbreviations

ATP: adenosine triphosphate
CGRP: calcitonin gene-related peptide
CT3: connective tissue group III muscle afferent
DMSO: dimethyl sulphoxide
GAPDH: Glyceraldehyde 3-phosphate dehydrogenase
GPR81: G protein-coupled receptor 81
HCAR1: hydroxyl carboxylic acid receptor 1
NF200: Neurofilament 200
PBS: phosphate-buffered saline.
